# Winter distribution of juvenile and sub-adult male Antarctic fur seals (*Arctocephalus gazella*) along the western Antarctic Peninsula

**DOI:** 10.1038/s41598-021-01700-w

**Published:** 2021-11-15

**Authors:** David March, Massimiliano Drago, Manel Gazo, Mariluz Parga, Diego Rita, Luis Cardona

**Affiliations:** 1 IRBio and Department of Evolutionary Biology, Ecology and Environmental Science, Faculty of Biology, University of Barcelona, Avinguda Diagonal 643, 08028 Barcelona, Spain; 2 Centre for Ecology and Conservation, College of Life and Environmental Sciences, University of Exeter, Penryn Campus, Penryn, TR10 9FE UK; 3 SUBMON - Marine Environmental Services, Ortigosa 14, 08003 Barcelona, Spain

**Keywords:** Animal migration, Conservation biology, Ecological modelling, Marine biology

## Abstract

Detailed knowledge of habitat use by marine megafauna is critical to understand their ecological roles and for the adequate management of marine resources. Antarctic fur seals (*Arctocephalus gazella*) inhabiting the Atlantic sector of the Southern Ocean prey largely on Antarctic krill (*Euphausia superba*) and play a central role in managing the krill fishery. Here, we assessed the demographic structure of three post-mating, early moult male haul-outs in the South Shetland Islands in early March and calculated the relative contribution of juveniles (1–4 years old) and sub-adult males (5–6 years) to the population remaining in maritime Antarctica after the breeding season. We also satellite tagged 11 juvenile males and four sub-adult males to analyze their movements and develop a species distribution model including both age classes. Our results highlighted the dominance of young individuals in the male population, revealed that they do not behave as central place foragers and identified key environmental drivers that affected their distribution at-sea throughout winter. Predicted potential foraging habitat overlapped highly with the known distribution of Antarctic krill, and identified the waters off the western Antarctic Peninsula and the Scotia Sea as the core of the distribution area of juvenile and sub-adult male Antarctic fur seals in winter. This pattern is similar to that of adult males but totally different from that of adult females, as the latter overwinter in areas at latitude 45–55° S. This segregation has implications for the ecology and management of the krill fishery.

## Introduction

Polar marine ecosystems are unique because of their extremely high degree of seasonality, low temperatures, strong oceanic currents and extensive seasonal sea ice cover^1,2^. Short days, extensive sea ice cover and the accumulation of snow on the ice severely limit primary productivity in winter, whereas dense phytoplankton populations develop in summer, as sea ice breaks up and melts. Increased summer primary productivity triggers the arrival of migratory seabirds and marine mammals, which in Antarctica prey largely on Antarctic krill (*Euphausia superba*)^1,3^. Nevertheless, krill abundance decreases dramatically in winter in most areas^4–6^, and most warm-blooded krill predators leave Antarctica at that time^7^. Only penguins, crabeater seals (*Lobodon carcinophaga*) and Antarctic fur seals (*Arctocephalus gazella*) remain in large numbers during winter in maritime Antarctica (i.e. the part of the Southern Ocean closer to the Antarctic continent and limited to the sea ice)^6,8–11^.

The Antarctic fur seal (*Arctocephalus gazella*) is the only polar species from the family Otariidae, although most of the breeding colonies are found in islands close to the Antarctic Polar Front^12^. Satellite tracking and stable isotope analysis demonstrated that the winter at-sea habitats of adult female Antarctic fur seals breeding both close to the Antarctic Polar Front and in the South Shetland Islands are usually located at latitude 45–55° S^13–21^. Models of habitat suitability for female Antarctic fur seals have identified oceanographic variables such sea surface temperature, sea surface height, wind velocity and the concentration of chlorophyll-a as the major habitat determinants, although their relevance varies idiosyncratically^17^ and distance to the colony is sometimes the major determinant of winter foraging grounds^20^.

Little is known about the winter habitat of male Antarctic fur seals and no habitat suitability model has been developed for them. The Antarctic fur seal is often qualified as ice-tolerant, but not ice-dependent^22^, and some authors have characterized adult males as ice-free oceanic foragers^23,24^. However, ship surveys have identified sea ice concentration as one of the major determinants of male Antarctic fur seal distribution off the South Shetland Islands and the Bransfield strait in winter^6^. Furthermore, males occuring at the South Orkney immediately after the breeding season remain in maritime Antarctica throughout winter^11^ and males breeding at islands close to the Antarctic Polar Front overwinter in maritime Antarctica^11,13–16,21,23,24^, which they do first from 2 to 3 years old^21,25^. Antarctic fur seals are highly sexually dimorphic^26–30^ and differences between sexes in winter habitat use could be related to differences in body mass, which in turn is a major determinant of their thermoregulatory skills and diving performance^31,32^. The study of juvenile and immature males, whose body mass is in between that of adult females and adult males, can be particularly useful to improve our understanding of habitat use by male Antarctic fur seals in winter.

Antarctic krill (*Euphausia superba*) is the staple food of Antarctic fur seals in the Atlantic sector of the Southern Ocean and the western Antarctic Peninsula^33–40^ and there is an urgent need to better understand the interactions between krill, their predators and the krill fisheries^3^. As already reported, most of the research on the spatial ecology of Antarctic fur seals has focused on females and the environmental monitoring program of the Commission for the Conservation of Antarctic Marine Living Resources (CCAMLR) focuses exclusively on females and their pups. However, juvenile and sub-adult males are the age classes with the highest krill consumption of the overall population^41^ and are therefore of particular interest for the management of the krill fishery.

Here, we study the population structure at three male haul-outs at Deception Island (South Shetland Islands), to assess the relative contribution of juveniles and sub-adults to the overall male population remaining in Antarctica after the breeding season. Furthermore, we use the data from 15 satellite tagged juvenile and sub-adult male Antarctic fur seals to develop a species distribution model and identify potential environmental drivers of their habitat use.

## Methods

### Study area

Fieldwork was done at Deception Island (62.963 S, 60.624 W), which hosts several major haul-outs used by several thousands of male Antarctic fur seals at a time. Sampling was carried out during the 2019 austral summer season, from late-February to early-March. Males arrive at Deception Island for their annual moult in mid-February, once the breeding season at Cape Shirreff (Livingston Island, South Shetland Islands) is over, and last until late March.

### Demographic structure at haul-outs

**Figure 1:**
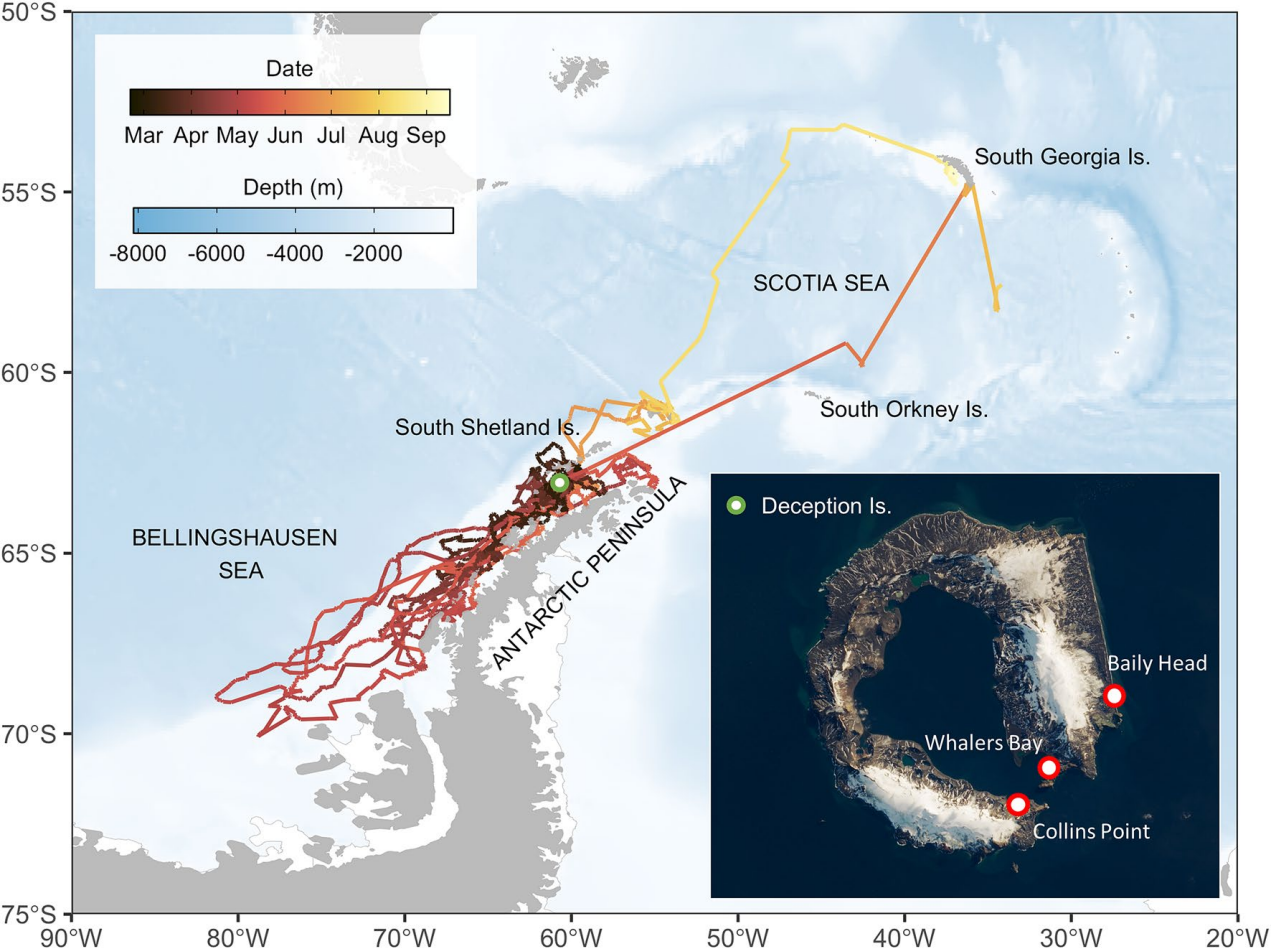
Study area and individual trajectories of juvenile and sub-adult male Antarctic fur seals. Deception Islands is denoted by the green symbol. Map inset shows the three haul-out locations where transect surveys were conducted at Deception Island (Satellite imagery from Landsat 8). Antarctic male fur seals were captured and instrumented at Collins Point. Map was generated using R version 4.0.2 (https://www.r-project.org/). NASA Earth Observatory image by Lauren Dauphin, using Landsat data from the U.S. Geological Survey.

We completed two visual censuses at three haul-outs (Fig. 1). Seven 10 m wide, parallel transects were done in each census at each haul-out. Transects run from the innermost part of the beach to the shore and transect length ranged from 15 to 230 m, depending on beach morphology. Transects were spaced 50 m apart. All seals in each transect were counted, sexed and classified as juveniles, sub-adults or adults according to body size and pelage coloration^42^.

### Animal capture and instrumentation

Antarctic fur seals were captured from February 20th to March 2nd, 2019 at Collins Point (Fig. 1) and instrumented with satellite tag linked platform terminal transmitters (PPTs) KiwiSat STANDARD series (model K2G 276A with wet/dry sensor; size: 78 × 43 × 27 mm, weight: 95 g) or KiwiSat DIVE series (model K2G 276A with depth sensor; size: 78 × 43 × 27 mm, weight: 95 g), manufactured by Sirtrack (Havelock North, New Zealand). KiwiSat STANDARD PTTs (n = 10) collected and transmitted location using ARGOS satellite service. KiwiSat DIVE PTTs (n = 5) also recorded dive data. The duty cycle was 24 h on every day and tags did not stop transmitting when hauled out. Juvenile males (n = 11) were captured using a hoop net and restrained in the same net while instrumented. The largest, sub-adult males (n = 4) were chemically restrained with a combination of midazolam (a benzodiazepine with sedative or tranquilizing action) and butorphanol (a synthetic agonist–antagonist opioid with analgesic action) remotely administered using a dart (5 ml) shot by means of a CO_2_ Dan-Inject JM rifle (Børkop, Denmark), with a dosage of 0.3 mg/kg and 0.2 mg/kg, respectively^43–45^. We chose this combination of drugs because both midazolam and butorphanol can be fully reversed with flumazenil and naloxone, respectively, and have a wide safety margin^44,46^.

Each individual was measured (nose to tail) and fitted with a PTT glued to the fur on the mid dorsal region between the scapulae, using AralditeTM quick set epoxy resin^13^. The entire operation lasted less than 18 min, from immobilization to release. Chemically restrained animals were also weighed on a scale, injected with the corresponding reversal agents (dosage flumazenil 0.003 mg/kg and naloxone 0.01 mg/kg) intramuscularly in the gluteal region, and released into a cage and monitored until they recovered. Details of the instrumented individuals are shown in Table 1.

**Table 1:** Animal tagging data. Trip duration is reported as mean and range, in days.

ID	Standard length (cm)	Weight (kg)	Tag type	Deployment date	Track duration (days)	Trips	Trip duration
64487	148	60	Standard	20/02/2019	108	22	2.4 (0.1–17.4)
64488	142	–	Standard	02/03/2019	197	25	1.2 (0.1–30.9)
64490	145	–	Standard	27/02/2019	124	29	0.5 (0.1–23.9)
64491	137	62	Standard	21/02/2019	41	15	0.4 (0.1–4,5)
64492	152	65	Standard	21/02/2019	42	7	3.2 (0.1–13.6)
64515	140	–	Standard	26/02/2019	26	7	1.6 (0.4–2.8)
64519	146	58	Standard	20/02/2019	102	24	0.5 (0.2–28.7)
64520	132	–	Standard	26/02/2019	49	12	1.0 (0.2–7.5)
64525	137	–	Standard	27/02/2019	33	7	2.6 (0.1–5.8)
64527	141	–	Standard	05/03/2019	154	22	1.6 (0.1–27.4)
64528	139	–	Dive	01/03/2019	72	21	0.6 (0.2–19.4)
64537	133	–	Dive	01/03/2019	84	13	2.6 (0.1–36.6)
64529	145	–	Dive	28/02/2019	2	0	0
64538	141	–	Dive	01/03/2019	30	16	0.4 (0.1, 3.1)
64555	128	–	Dive	28/02/2019	78	23	1.5 (0.1–14.4)

### Foraging trip and dive analysis

Pearson’s correlation coefficient was used to explore the relationship between the duration of the foraging trips and daylength. A General Linear Mixed Model (GLMM), with month as a fixed effect and individual seal identity as a random effect, was used to assess the existence of monthly changes in the deepest daily dive, from March to May, of the four seals instrumented with depth sensors. IBM SPSS v. 25 was used for both analyses.

### Location data processing

Individual seal trajectories were visually inspected to identify haul-out events and trim tracks into individual trips (i.e. ocean tracks in between haul-outs). Near-duplicate positions, defined as animal positions that occurred 2 min or less after an existing position fix from the same animal, were removed^47^. Argos data were then filtered using a speed, distance and angle filter^48^ that removed all location class Z values and points with unrealistic swimming speeds (> 3 m s^−1^)^49,50^ or unlikely turning angles (all spikes with angles smaller than 15 or 25 degrees were removed if their lengths were greater than 2.5 or 5 km, respectively) using the “argosfilter” R package^48^. Tracks with data gaps in excess of 7 days were broken up for separate modelling (i.e. each portion of the track was treated independently). A state-space model (SSM) was used to estimate locations at regular time intervals (6 h) and account for measurement error in the original observations using the “foieGras” R package^51,52^. We fitted the SSM using a correlated random walk model with the Template Model Building (TMB) for fast estimation. After checking for convergence, all tracks were retained for further analyses.

### Pseudo-absences

Satellite tracks represent presence only data. In order to use a binomial response in the habitat model (i.e. presence and absence), we followed a two-step approach to generate pseudo-absences. First, we generated simulated tracks to represent the available habitat (i.e. where the animals could go given their movement characteristics and duration of the track). For each real track, we simulated 50 pseudo‐tracks by fitting a first‐order vector autoregressive model characterized by the step lengths and turning characteristics of the observed track^19,23^. The number of simulations was selected as a compromise between computational cost and the amount of generated locations for further random sampling (see habitat model section). Simulations were generated using the “availability” R package (https://github.com/AustralianAntarcticDivision/availability). For each simulation, we fixed the initial location (i.e. the first track location) and restricted the following locations to the sea by defining a custom land mask of the study area using the GEBCO bathymetry (www.gebco.net). Such simulations recreate the movement characteristics of the original tracks, taking into account their autocorrelations structure^53^, but are independent of the underlying environment. However, simulated tracks can generate replication at the same locations of the real track, hence leading to contradictory information in binomial models (i.e. same location and date defined as either presence and absence) and potentially reduce model performance^54^. To reduce the amount of pseudo-replication and prevent overlap between real and simulated tracks, we gridded all presence and pseudo-absence locations per individual at 0.1 degrees on a daily basis and filtered out pseudo-absences that were adjacent to any presence grid cell (i.e. all individuals considered) within a temporal window of 2 days.

### Environmental data

A set of 13 environmental covariates were matched to estimated locations and pseudo-absences to analyze the habitat use of Antarctic fur seals (Table 2, Supplementary Fig. S1). Covariates were chosen on the basis of biological relevance and spatial and temporal resolution. Two static variables included bathymetry (GEBCO 2014 Grid, https://www.gebco.net/), and slope of the seabed. Bathymetry determines whether the water column can be stratified and whether air-breathing predators can access the seabed, whereas the slope of the seabed has a strong influence on current velocity and direction and processes such upwelling. Dynamic variables were sourced from daily fields from physical and biogeochemical data-assimilative numerical models (Copernicus Marine Environment Monitoring Service, https://marine.copernicus.eu/). Sea surface temperature and salinity are useful to characterize distinct water masses and sea surface temperature is also critical for thermoregulation. We derived their gradients, as they can help to identify transition areas between distinct water masses. Sea ice fraction, distance to the ice edge and sea ice thickness determine largely the accessibility of air-breathing marine mammals to the seasonal sea-ice region and its associated prey. The sea ice limit was set where the sea ice fraction was 15%^55,56^. Sea surface height and eddy kinetic energy are proxies for the intensity of mesoscale activity. Dynamic variables included the mixed layer depth, with a critical role in determining vertical mixing and the intensity of primary productivity. Chlorophyll-a concentration was a proxy for primary productivity. Chlorophyll-a concentration and eddy kinetic energy were highly right skewed and were log (x + 1) transformed prior to analysis. All covariates were bilinearly interpolated to a common extent (i.e. encompassing all observed and simulated tracks) and resolution (0.1° × 0.1° pixel size). Each presence and absence location was temporally (i.e. same day) and spatially matched to environmental data by averaging their values within a 15 km radius, hence accounting for uncertainty in covariate data arising from observation error and filling missing data. Across all covariates, there were only a few missing values in chlorophyll-a concentration (0.63%); thus we retained all covariates for further analyses.

**Table 2:** Environmental variables used as predictors in the habitat suitability model.

Abbre-viation	Description	Unit	Spatial resolution	Temporal resolution	Data source/calculation method
BAT	Bathymetry	m	0.0083°	Static	Extracted from GEBCO 2014 Grid, https://www.gebco.net/
SLP	Slope	°	0.0083°	Static	Derived from BAT calculating the slope with the “terrain” function in the “raster” R package
SIC	Sea ice area fraction	1	0.083°	Daily	Extracted from the Global Ocean Sea Physical Analysis and Forecasting Product https://marine.copernicus.eu/
EDGE	Distance to ice edge	km	0.083°	Daily	Derived from SIC after calculating distance to sea ice concentration > 15%, using the “gridDistance” function in “raster” R package
SIT	Sea ice thickness	m	0.083°	Daily	Extracted from the Global Ocean Sea Physical Analysis and Forecasting Product https://marine.copernicus.eu/
SST	Sea surface temperature	°C	0.083°	Daily	Extracted from the Global Ocean Sea Physical Analysis and Forecasting Product https://marine.copernicus.eu/
SSTg	Sea surface temperature gradient	°	0.083°	Daily	Derived from SST calculating the slope with the “terrain” function in the “raster” R package
SAL	Salinity	PSU	0.083°	Daily	Extracted from the Global Ocean Sea Physical Analysis and Forecasting Product https://marine.copernicus.eu/
SALg	Salinity gradient	°	0.083°	Daily	Derived from SAL calculating the slope with the “terrain” function in the “raster” R package
SSH	Sea surface height	m	0.083°	Daily	Extracted from the Global Ocean Sea Physical Analysis and Forecasting Product https://marine.copernicus.eu/
EKE	Eddy kinetic energy	m^2^ s^-2^	0.083°	Daily	Derived from sea water velocity extracted from Global Ocean Sea Physical Analysis and Forecasting Product as EKE = 0.5(U^2^ + V^2^)
CHL	Chlorophyll a concentration	mg m^-3^	0.25°	Daily	Extracted from the Global Biogeochemical Analysis and Forecasting Product https://marine.copernicus.eu/
MLD	Mixed layer depth	m	0.083°	Daily	Extracted from the Global Ocean Sea Physical Analysis and Forecasting Product https://marine.copernicus.eu/

### Habitat suitability model

We developed a species distribution model using boosted regression trees (BRT), a machine-learning method commonly used to model animal tracking data^57,58^. BRT performs better using the same number of pseudo-absences as available presences; we used a stratified random subsampling of pseudo-absence data to select the same number as presence observations per day and per individual. This 1:1 ratio was recommended for machine-learning methods^59^, and applied in species distribution models of animal telemetry^53,57^. Although collinearity between environmental variables does not affect BRT predictions, it can affect the interpretation of the model^60^. Therefore, we assessed collinearity among variables calculating the Spearman pairwise correlation coefficient. Most predictors were uncorrelated (Spearman correlations < 0.7) and only sea ice thickness (highly correlated with sea ice fraction) was discarded from further analysis (Supplementary Fig. S2).

We used the “dismo” package in R^61^ to fit the BRT using a Bernoulli family, appropriate to the response variable of presence (1) and absence (0). BRT requires the optimization of four parameters^62^: the number of trees (boosting iterations), tree complexity, the learning rate (shrinkage) and the bag fraction (proportion of data randomly selected at each iteration). We created combinations for potential values: number of trees = 50–10,000 in 50 tree increments; tree complexity = 1, 3 or 5; learning rate = 0.005, 0.001, 0.05, 0.01; and bag fraction = 0.5, 0.6 or 0.7. Following previous recommendations^62^, we selected the combination with > 1000 trees that minimized the area under the receiver operating characteristic curve (AUC, a measure of model predictive performance) during cross-validation. In case of ties, we prioritized models with larger learning rates, smaller tree complexities and fewer number of trees to reduce overfitting. In order to account for the repeated-measures structure derived from telemetry data, we incorporated a block factor in the cross-validation process^19,63^. We used individual seals as folds in a leave-one-out cross-validation, meaning that all data from a given seal (both observed and simulated locations) were excluded from the training dataset and used to validate the model. After running the parameter optimization (Supplementary Table S1), we selected these parameters (number of trees = 1050, tree complexity = 5, learning rate = 0.01, bag fraction = 0.5) to fit the final model. Variable selection in BRT is achieved because the model largely ignores non-informative predictors when fitting trees^62^. However, to drop unimportant variables the model included an additional variable, with a random number between 1 and 100, to serve as an indicator for variables that have influence greater or less than random^64^. All environmental variables had influence greater than the random number variable and were included in the final model. Finally, we used the fitted model to generate spatial predictions of the habitat suitability for the entire study region on a daily basis. To account for model stochasticity and estimate the uncertainty associated with these predictions, we used a bootstrap approach^57,62^. We fitted the model 50 times by sampling half the data (with replacement) to map daily predictions, using the median, for the study region^58^. As a measure of uncertainty, we calculated the 95% confidence interval range of the 50 values in each cell. In order to summarize seasonal trends, we generated monthly averages from daily predictions.

### Accessibility model

The modelling approach described above estimates habitat suitability of a given location based on its environmental characteristics. However, it does not consider the accessibility of a given cell. Following previous works, we used a second set of models to account for this factor^19,58^. Given that tagged individuals did not behave as central place foragers (see “Results” section), we modelled accessibility of a given grid cell as a function of distance beyond the ice edge (15% ice concentration) using a binary response^58^: accessible (1) (i.e., cells with any observed or simulated location), non-accessible (0) (i.e., cells with no observed or simulated locations). We fitted binomial models with a smooth, monotonic decreasing constraint using the “scam” R package^65^, under the assumption that accessibility should decrease with the distance to the ice edge. We also assumed that sea ice concentrations > 15% were not accessible to Antarctic fur seals. Similarly to the habitat model, we used a bootstrap approach to account for model uncertainty and fitted the model 50 times by sampling half the data (with replacement). Predictions from the habitat suitability were then weighted by the predictions of the accessibility model.

### Code availability

All analyses and plots were undertaken using the R programming language^66^. The code will be made available upon publication at Github (https://github.com/dmarch/agazella).

### Ethics statement

All animal handling procedures in this study were reviewed and approved by the Ethics Committee in Animal Experimentation of the University of Barcelona and the Government of Catalonia (project No 10292) in accordance with relevant guidelines and regulations. The procedures adhered to the ARRIVE guidelines and requirements of the ethics committee of the Spanish Polar Institute that approved all our fieldwork under the permit No: CPE-2018-4.

## Results

### Demographic structure

**Figure 2:**
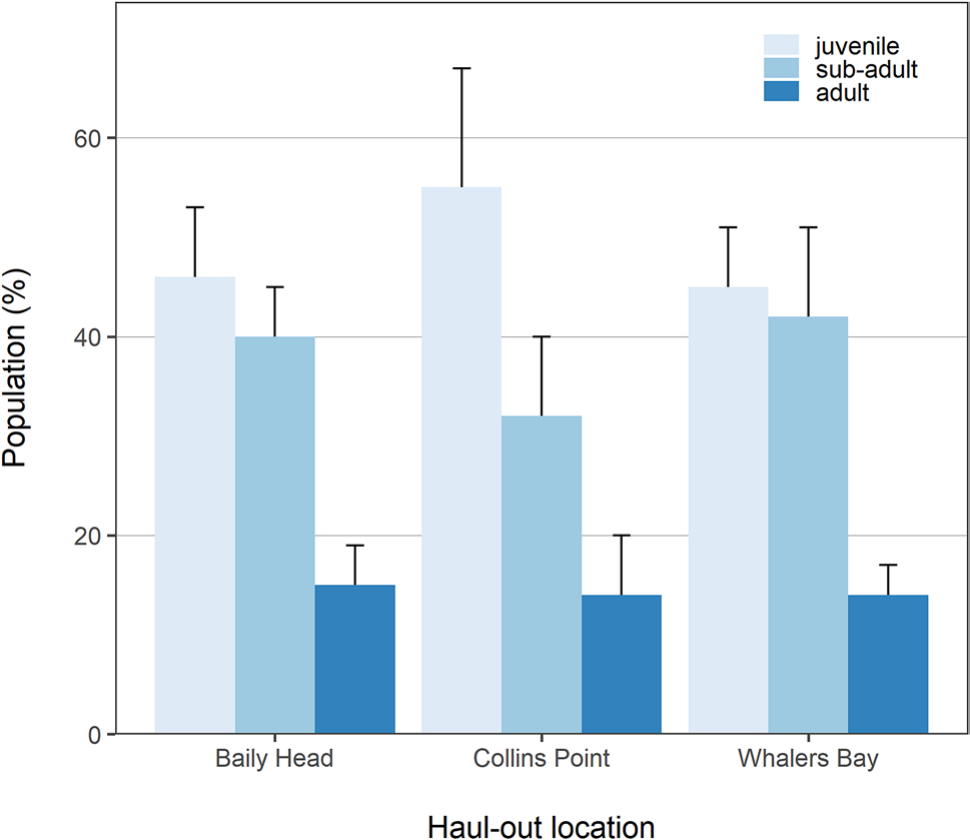
Demographic structure of the male Antarctic fur seal at haul-outs in Deception Island in early March 2019. Vertical bars denote standard deviation of two samples. Total number of seals: Baily Head: 103, Collins Point: 281, Whalers Bay: 158.

A total of 542 male fur seals were counted in the surveys, resulting in an average density of 99.3 ± 34.5 seal ha^−1^. Adults represented 13.7 ± 2.4% of the surveyed animals, whereas sub-adults contributed 36.5 ± 8.0% and juveniles 49.7 ± 8.7% to the total count (mean ± SD). The population make-up was similar at the three haul-out sites (Fig. 2).

### Seal movements

The instrumented male Antarctic fur seals ranged 128–152 cm in length (Table 1) and were likely 2–5 years old^26^. One of the KiwiSat DIVE PTTs (#64529) transmitted for only 48 h and was not considered in further analyses. The other 14 PPTs transmitted for 26–197 days (Table 1) and most of the seals were tracked through early (March–May) and mid-winter (June–August). During that period, their movements ranged from the northern part of the Bellingshausen Sea, to the south, to the South Georgia Island, to the north (Fig. 1 and Supplementary Fig. S3).

**Figure 3:**
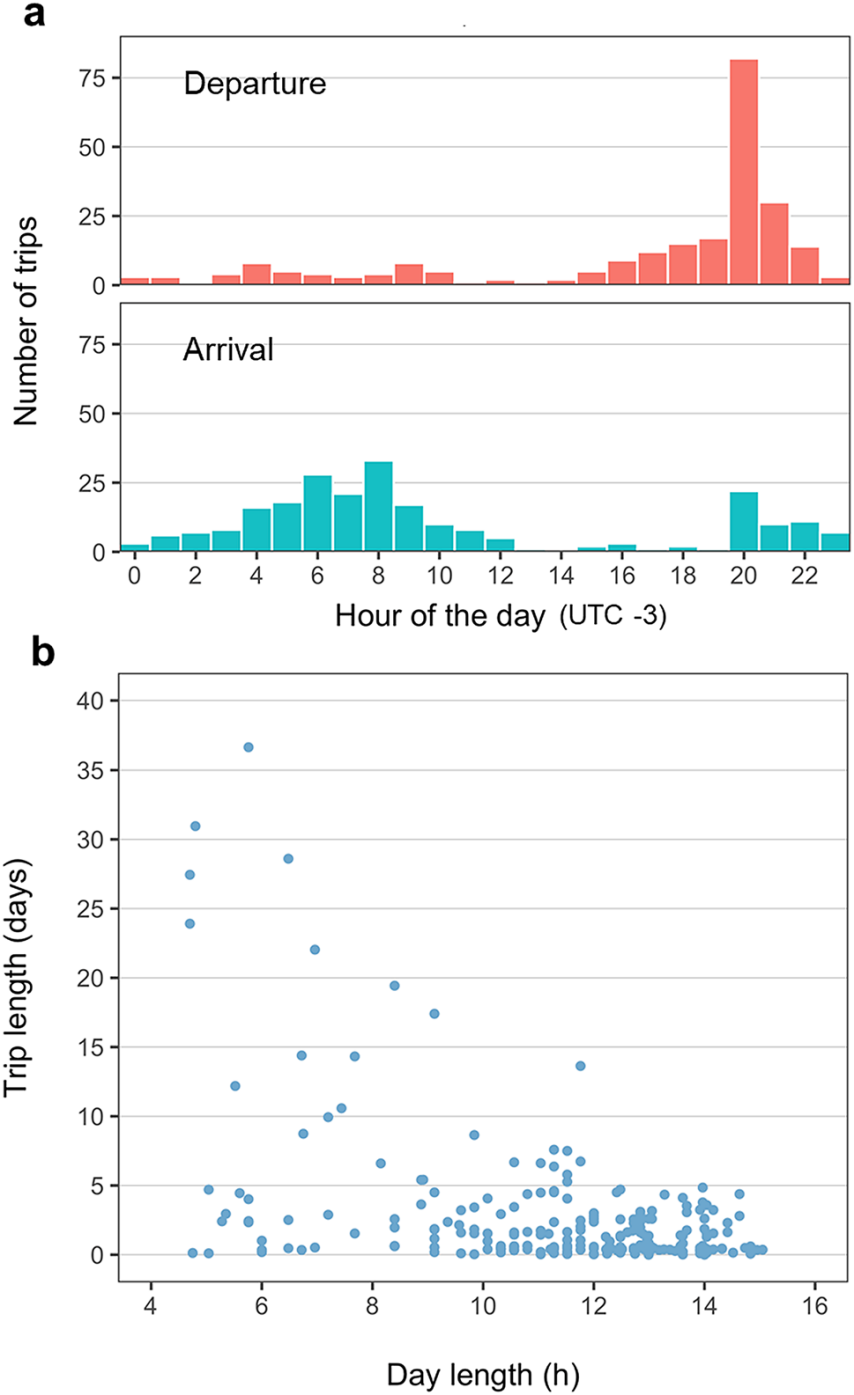
Foraging trips of juvenile and sub-adult male Antarctic fur seals. (**a**) Histogram of departure and arrival times. (**b**) Relationship between day length and trip length.

The instrumented seals departed from haul-out sites in the evening and hauled-out again in the morning (Fig. 3a), but they seldom engaged in round trips, as they returned to the same haul-out only in 35 out of 243 trips (Supplementary Fig. S4). Until March 21st (fall equinox), most trips were nocturnal and lasted less than 12 h, although trips lasting up to 5 days were not uncommon (Fig. 3b; Supplementary Fig. S4). Trips lasting a single night became uncommon and trips lasting more than 10 days increased in frequency after the fall equinox (Fig. 3b and Supplementary Fig. S4). As a result, the duration of the foraging trips was negatively correlated with daylength (r = − 0.472, *p* < 0.001, n = 243).

The 14 tagged male Antarctic fur seals remained off the South Shetland Islands or between the archipelago and the western Antarctic Peninsula (i.e. the Bransfield Strait) until mid-March, when five of them headed south (Fig. 1). Two other individuals moved south in mid-April. As a result, six of the eight instrumented Antarctic fur seals with active PTTs on May 1st have reached Marguerite Bay or the northern limit of the Bellingshausen sea and only two other individuals remained in the Bransfield Strait. Nevertheless, the individuals foraging off Marguerite Bay headed north during the first week of May, as sea ice spread into the area. They reached the Bransfield Strait on June 1st, when one of the individuals, which had remained there since tagging, headed to the South Orkney Islands. After a few days off the South Orkney Islands, the specimen moved to the South Georgia Islands, traveling back south to the sea ice limit in early August. Finally, the last two individuals left the Bransfield strait immediately after the winter solstice, heading to Elephant Island. One of them moved later to the South Georgia Islands where it remained until September 2019. No obvious differences were observed between the movement patterns of sub-adults and juveniles (Supplementary Fig. S3): one sub-adult (#64,491) and five juveniles (#64515, 64525, 64527, 64538 and 64520) remained within the Bransfield Strait, two sub-adults (#64487 and 64519) and three juveniles (#64488, 64528 and 64537) engaged in a round migration from the Bransfield Strait to Marguerite Bay and the northern Bellingshausen sea and one sub-adult (#64492) and two juveniles (#64555 and 64492) moved south after tagging but transmission ceased before they reached the northern Bellingshausen Sea. As a result, we modelled juveniles and sub-adults together.

### Seal dives

**Figure 4:**
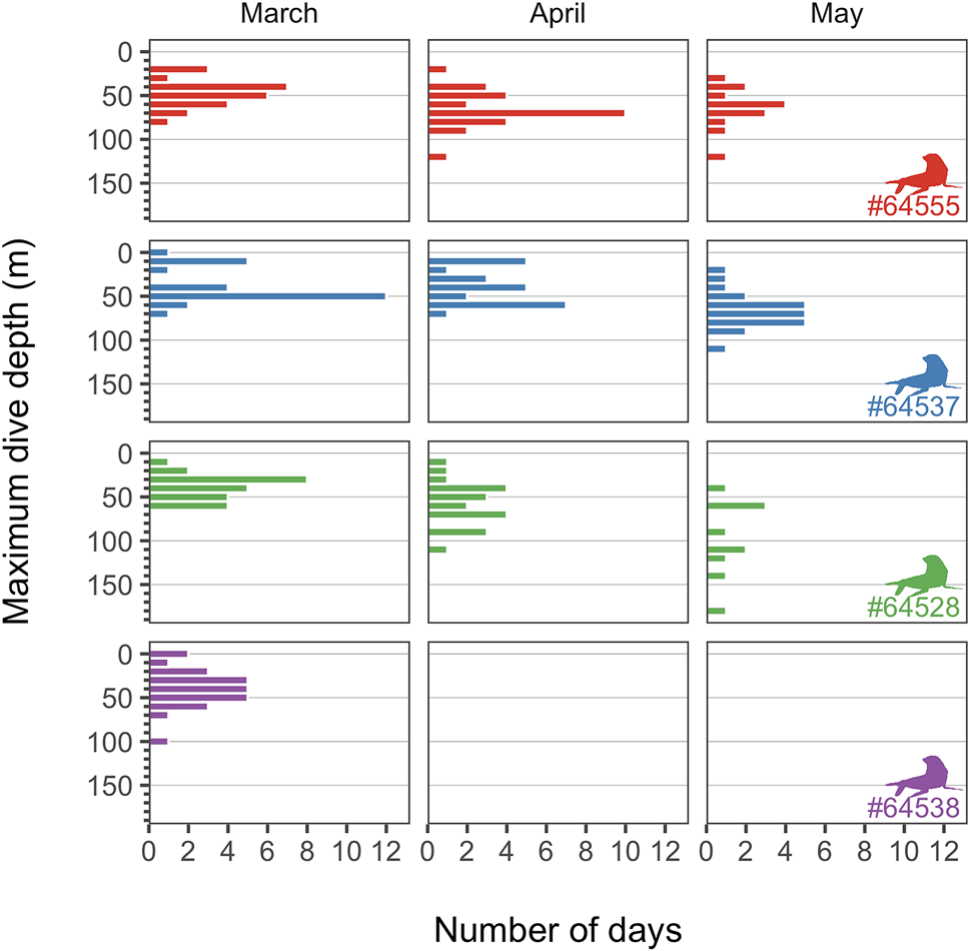
Daily maximum depth of four juvenile male Antarctic fur seals instrumented with depth sensors.

None of the four juvenile male Antarctic fur seals instrumented with DIVE PTTs was tracked beyond mid-May. The deepest recorded dive was 180 m, although the average depth of the deepest daily dive of the four tracked individuals increased significantly as winter advanced: 40.7 ± 3.4 m in March, 54.0 ± 13.4 m in April and 72.5 ± 19.3 m in May (Fig. 4; GLMM: month, df = 2, Chi-square = 53.081, *p* < 0.001; seal identity: df = 1, Chi-square = 5.73, *p* < 0.017). Variability between individual seals explained 17% of the observed variance.

### Habitat suitability

**Figure 5:**
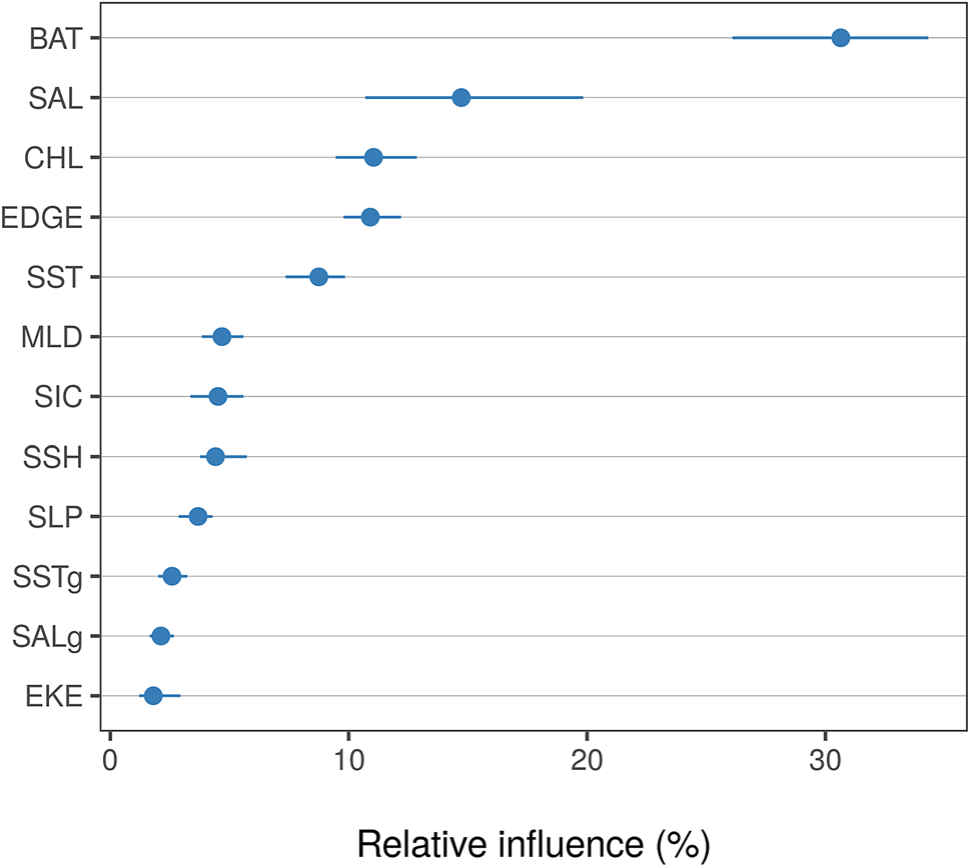
Relative influence of environmental variables used to model the habitat suitability of juvenile and sub-adult Antarctic fur seals. Dots represent medians and lines represent the 95% confidence interval range of the bootstrap predictions (n = 50). BAT: bathymetry, SAL: salinity, CHL: log-transformed (x + 1) concentration of chlorophyll-a, EDGE: distance from the sea ice limit, SST: sea surface temperature, SIC: sea ice fraction, SSH: sea surface height, MLD: mixed layer depth, SLP: slope, SSTg: sea surface temperature gradient, SALg: salinity gradient, EKE: eddy kinetic energy.

**Figure 6:**
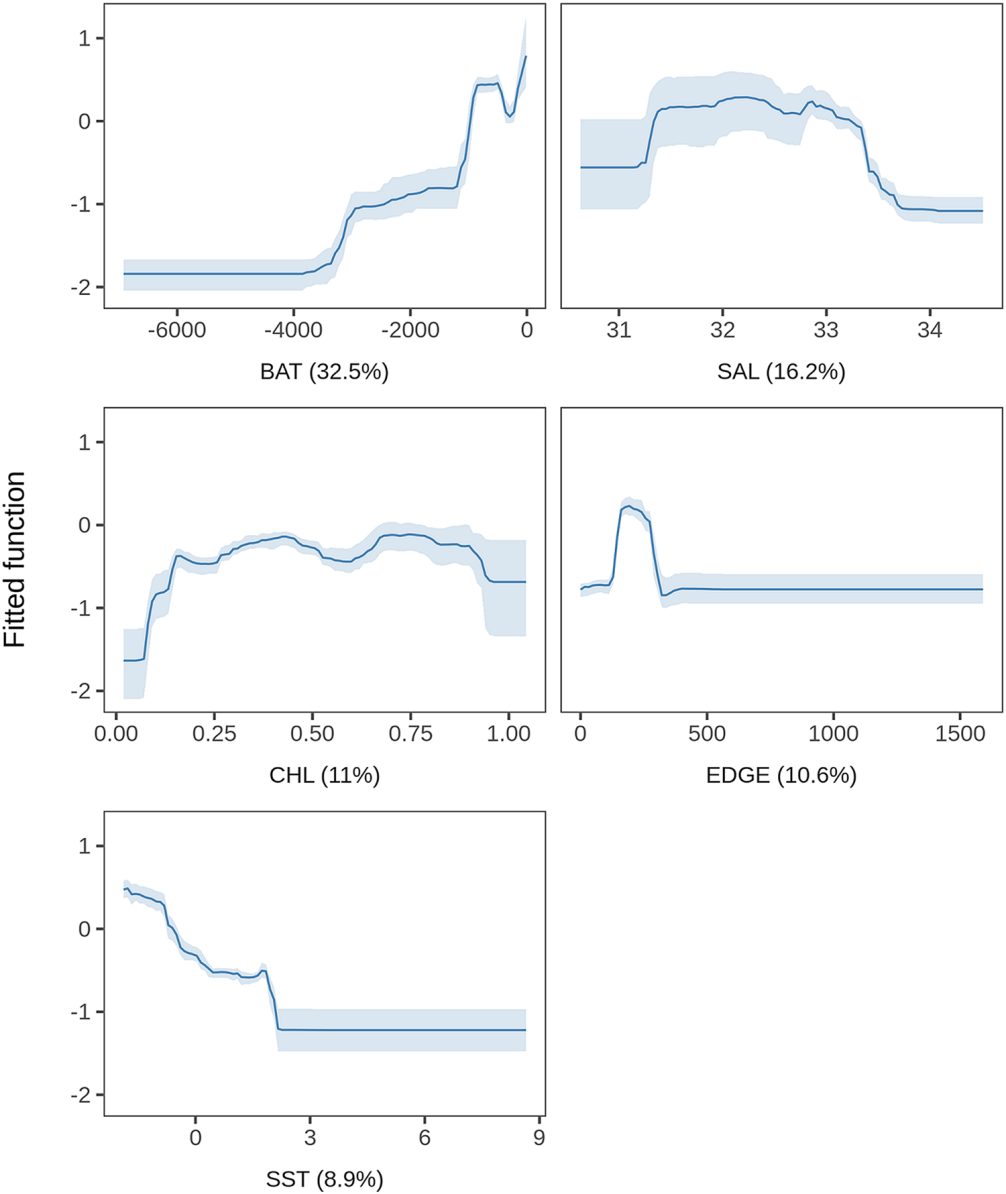
Partial dependence plots of the top five variables from the BRT model. Relative contribution in percentage is provided between parentheses for each variable. BAT: bathymetry, SAL: salinity, CHL: log-transformed (x + 1) concentration of chlorophyll-a, EDGE: distance from the sea ice limit, SST: sea surface temperature. The shading shows the 95% confidence intervals estimated from 50 bootstrap samples of the data set.

**Figure 7:**
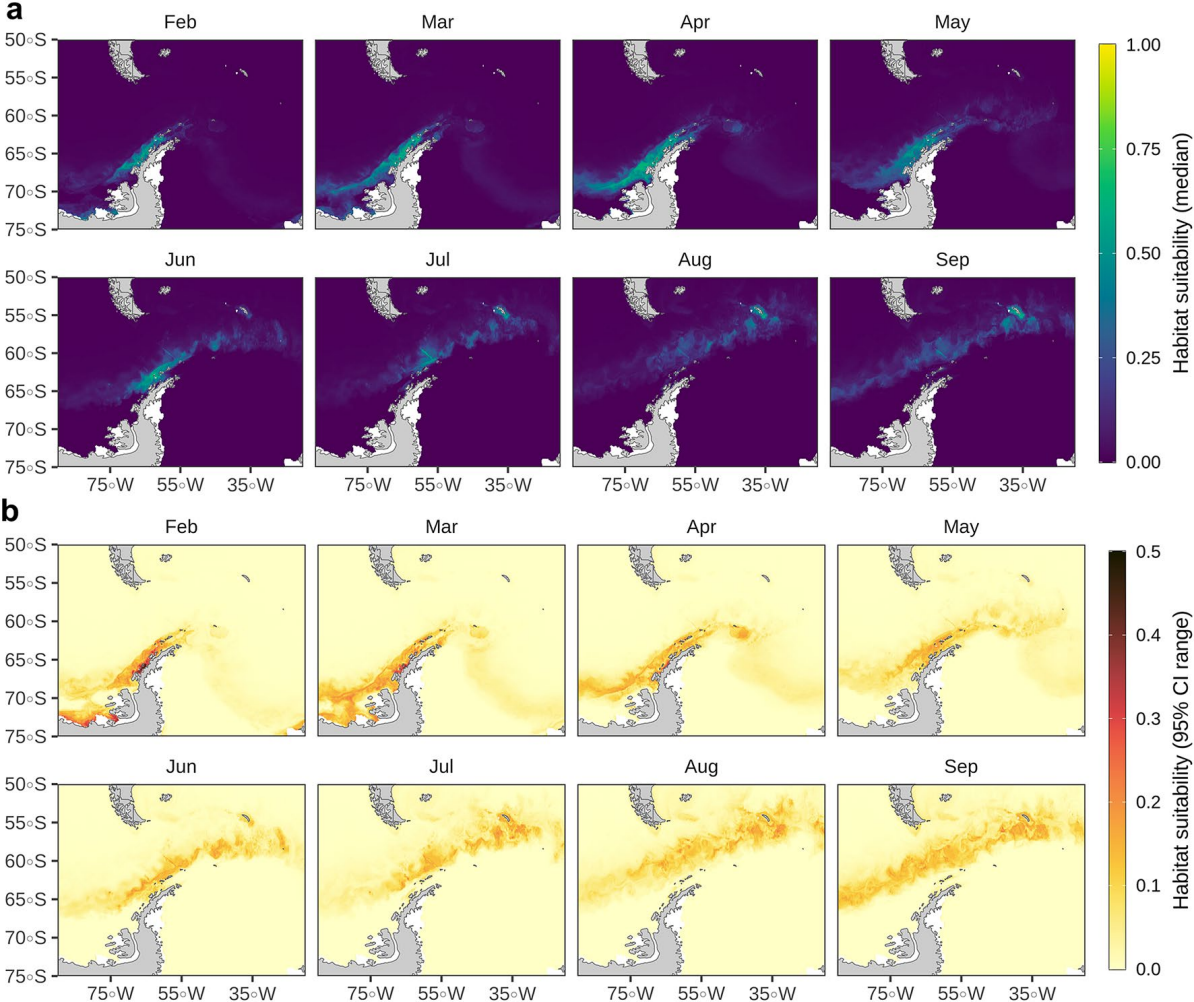
Predicted habitat suitability of juvenile and sub-adult Antarctic fur seals for February—September 2019. (**a**) Monthly average of daily predictions (median of the bootstrap predictions, n = 50). (**b**) Monthly average of daily uncertainty estimates (95% confidence interval range of the bootstrap predictions, n = 50). Maps were generated using R version 4.0.2 (https://www.r-project.org/).

Model assessment using cross-validation procedures suggested a good fit to the observed data, with the model explaining 38% of the cross-validated deviance and having a high predictive performance (cross-validated AUC score = 0.88; Supplementary Table S1). See Table 2 for variable definition and their acronyms. A static variable (BAT) and a set of dynamic variables (SAL, CHL, EDGE, and SST) were found amongst the top predictors (Fig. 5), exhibiting non-linear response curves (Fig. 6). Conversely, derived gradients (SSTg, SALg) and indicators of mesoscale activity (EKE) were weaker predictors. According to the model, the tagged juvenile and subadult males preferred areas less than 1000 m deep at 200–250 km from the ice edge, with low salinity (31–33 PSU), and very cold water (SST < 2 °C). Predicted habitat suitability averages on a monthly basis (February-September) together with their uncertainty estimates are shown in Fig. 7. Predicted habitat suitability changed seasonally, showing a southward expansion towards the Bellingshausen Sea during April and May, followed by a northward expansion towards the South Georgia Islands from June onwards.

## Discussion

This work provides new insights from the early life stages of Antarctic fur seals, a keystone species in the Southern Ocean and major consumer of Antarctic krill. Here, using visual surveys and satellite telemetry we have assessed the demographic structure at haul-outs after the breeding season and analysed the habitat use of juvenile and sub-adult males present in maritime Antarctica in winter. Our results highlighted the dominance of these early life stages in the male population and identified key environmental drivers that affect their distribution at-sea. Such contributions could benefit marine conservation in Antarctica, with a major interest for the management of the krill fishery.

Sea surface temperature, bathymetry, primary productivity, wind speed and distance to the ice edge are often the most important predictors of habitat preference by air-breathing marine predators in the Southern Ocean, although the relative importance of each parameter varies across species^10,18–20,58^. Consistently, our modelling approach identified bathymetry, sea surface temperature, salinity, concentration of chlorophyll-a, and distance to the ice edge as the most relevant predictors of habitat preference for juvenile and sub-adult male Antarctic fur seals. In late summer and early winter, suitable habitat spanned along the western Antarctic Peninsula, from the Bransfield Strait to the Bellingshausen Sea. In mid-winter and late-winter, suitable habitat displaced northward, from the South Georgia Islands to Adelaide Island. Overall, the habitat suitable for juvenile and sub-adult male Antarctic fur seals matched the known distribution of Antarctic krill^4,5,67^, which is their main prey in that sector of the Southern Ocean^33–40^.

Sea surface temperature and surface salinity play a relevant role in the model because they delineate successfully the water masses north and south to the Antarctic Polar Front (Supplementary Fig. S1). Likewise, bathymetry and distance to the ice edge had relevant contributions to the model because they characterized a region of open water immediately off the sea ice edge (Supplementary figure Fig. 1S). Finally, chlorophyll-a concentration was retained in the model because it is a major determinant of krill distribution. We did not incorporate distance to haul-out because juvenile and sub-adult male Antarctic fur seals did not behave as central place foragers during the tracking period, as demonstrated by the very low proportion of foraging trips departing and arriving to the same haul-out (Supplementary Fig. S4).

Model results suggest that Antarctic fur seals are strongly affiliated with both static (e.g. bathymetry) and dynamic (e.g. distance to ice edge) environmental variables. In particular, the association with dynamic variables reinforces using contemporaneous daily products from numerical models. Numerical models offer new opportunities to circumvent the limitation of in situ observations and satellite remote sensing (e.g. cloud cover, variable resolution, sub-surface data) and facilitate prediction across large dynamic seascapes^64,68,69^. However, numerical models can have errors, which should be considered when interpreting the results. For example, sea ice concentration is overestimated in Antarctica during austral winter and underestimated during austral summer in the model used here^70^, albeit the general dynamic is consistent with previous works^71^. Refinement of data-assimilative models in polar regions can be challenging due to undersampling from traditional observing platforms, but a new generation of ice-capable Argo floats and animal-borne sensors offers new opportunities for further enhancement of models^72,73^. In addition, two major caveats should be kept in mind when interpreting the results reported here. First, all individuals were tagged from the same location, thus biasing the study to individuals already present in the Bransfield Strait and from the same haul-out. Second, tag durations ranged widely, with only five individuals tracked beyond May and two beyond August, thus further biasing our results during mid and late winter.

Previous research based on ship surveys identified distance to land as a major determinant of at-sea fur seal density in the Bransfield Strait and adjoining areas during the summer months^74^. This is because male Antarctic fur seals haul-out frequently in summer at the South Shetland Islands to molt^75^ and make short, nocturnal foraging trips (Fig. 3a). However, our study indicates they haul-out only sporadically as winter advances (Fig. 3b). In fact, winter ship surveys identified sea-ice concentration and krill biomass as the major habitat determinants of habitat use during winter at the Bransfield strait^6^. This agrees with the habitat suitability model developed here, relying largely on the distance to the ice edge and bathymetry, chlorophyll-a concentration and sea surface temperature, the four latter strongly related to the distribution of Antarctic krill^4,5^.

According to our results, the South Shetland Islands and the Bransfield Strait represent the core of the distribution area of juvenile and sub-adult male Antarctic fur seals in the western Antarctic Peninsula in winter. This conclusion is in accordance with previous ship surveys^6,74^ and satellite telemetry data^11^, although previous ship surveys did not asses the abundance of Antarctic fur seals south to the South Shetland Islands and did not assess either sex or age class of the spotted individuals. The Bransfield Strait and the South Shetland Islands were indeed included in the list of Antarctic areas of ecological significance in a recent study^58^ and are also a hotspot for Antarctic krill year-round^4–6,67^.

The overall evidence demonstrates that juvenile, sub-adult and adult male Antarctic fur seals overwinter consistently in maritime Antarctica^13–16,19,21,23,24^. According to the evidence from stable isotope ratios in vibrissae, most male Antarctic fur seals born at islands close to the Antarctic Polar Front overwinter in maritime Antarctica for the first time when they are 2 or 3 years old^21,25^. Younger juveniles often exhibit distinct stable isotope ratios in their vibrissae^21,25^, either because they use foraging grounds at much lower latitude due to poorer thermoregulatory skills in very cold water^76^, they fast for a long period after weaning^77,78^ or both. There are no published stable isotope data from males born at the South Shetland Islands to date, but the data reported here and a recent study^11^ suggest that males present in the South Orkney and the South Shetland Islands immediately after the breeding season remain in maritime Antarctica throughout winter and likely occur in large numbers in the region year-round.

Antarctic fur seals are sexualy dimorphic, with adult males reaching 120–140 kg and adult females 25–50 kg^26–30^. A larger body mass improves thermoregulation in cold environments^31^ and the thermal insulation of adult male Antarctic fur seals is further improved by a thicker blubber compared to that of adult females (14 mm vs. 8 mm)^26^. Moreover, the body fat reserves of females might be depleted after the breeding season, thus turning females more sensitive to low sea surface temperature in early winter than in summer. If so, females might need to forage on energy rich prey at lower latitude to replenish their body fat reserves. However, thermoregulation is not necessarily the main reason why females do not overwinter in maritime Antarctica. Young males, which overwinter in maritime Antarctica (this study and previous studies^11,21,25^), have a blubber thickness similar to that of adult females^26^ and the sea surface temperature of the summer foraging grounds of the females breeding at the South Shetland Islands^17^ is not different from the sea surface temperature of the foraging grounds used throughout the present study by juvenile and subadult males.

Changes in the vertical distribution of Antarctic krill in winter could be the main reason why adult females do not overwinter in maritime Antarctica. The highest biomass of Antarctic krill in the whole Southern Ocean is observed at night in the top 50 m of the water column during the summer months^4,5^, which is why adult female Antarctic fur seals breeding in the South Georgia Islands, the South Shetland Islands and Buovetøya Island forage primarily at night during the breeding season and usually dive less than 50 m^27,32,79,80^. The aforementioned is also true for the juvenile males present in the Bransfield Strait in late summer and early winter (Figs. 3 and 4). On the contrary, adult males are more flexible and forage frequently deeper and during the day at South Georgia Islands in summer^32^. Increased body mass results in larger oxygen stores that allow adult male Antarctic fur seals diving deeper (100 m vs. 39 m) and longer (231 s vs. 83 s) than adult females and hence access the krill that moves to deeper water during the day^32^.

In winter, the biomass of Antarctic krill declines markedly and concentrates in shelf areas, usually deeper than 100 m^4–6,81^. Adult, sub-adult and juvenile males inhabiting the Atlantic sector of the Southern Ocean and the western Antarctic Peninsula respond by diving deeper as winter advances (Fig. 4 and a previous study^11^). However, Antarctic krill is mostly out of reach of adult female Antarctic fur seals in winter, as they seldom dive deeper than 100 m^29,30,32,50,80,82^. This would explain why females breeding at the South Shetland Islands migrate to northern foraging grounds and why those breeding in islands close to the Antarctic Polar front remain close to their breeding grounds in winter, contrary to males^13–21^. The possible role of intraspecific competition between sexes in a scenario of decreased krill availability remains to be tested.

Independently on the actual reason why adult females leave maritime Antarctica in winter, the year-round presence of males in the region suggests that they are responsible for most of the krill consumption by the whole Antarctic fur seal population in the Atlantic sector of the Southern Ocean^11^. Juvenile and sub-adult males are of particular interest because they prevail in the population at the South Georgia Islands^26,32,41^ and Deception Island (Fig. 2). A previous study^41^ demonstrated that juvenile and sub-adult males contributed to more than half the overall consumption of Antarctic krill by the South Georgia population. This suggests that juvenile and sub-adult Antarctic fur seals would be the major contributors to the overall consumption of Antarctic krill by the Antarctic fur seal population if the demographic structure of other major haul-outs in the South Orkney and South Shetland Islands matches that reported for Deception Island and the South Georgia islands.

The western Antarctic Peninsula and the Atlantic sector of the Southern Ocean are experiencing accelerated warming^83^, which will certainly impact the distribution of wildlife. Historically, the Scotia Sea supported a dense population of adult Antarctic krill due to the advection of larvae from the Antarctic Peninsula, with a secondary maximum in the Bransfield Strait^5,67^. This probably explains why the South Georgia Islands supported the largest breeding population of Antarctic fur seals during the second half of the twentieth century^12^. However, krill distribution in the Atlantic sector of the Southern Ocean has contracted southwards as a result of decline in the duration and extent of sea-ice and currently the highest krill abundance in summer is observed in the Bransfield Strait and Marguerite Bay^4,67^. This explains why the Bransfield Strait is currently the core of the distribution of juvenile, sub-adult and adult Antarctic fur seals in the Atlantic sector of the Southern Ocean (this study and previous work^11^). Declining abundance of Antarctic krill in the Scotia Sea has also resulted in a decrease in the reproductive success of female Antarctic fur seals breeding at the South Georgia Islands^84,85^. Such decline has not been balanced by improved reproductive success at the South Shetland Islands, because of an increased predation of pups by leopard seals^86^. Likewise, increased biomass of krill at Marguerite Bay is unlikely to be demographically relevant for Antarctic fur seals in the absence of any nearby colony, although juveniles and sub-adults exploit the area in late summer and early winter, as revealed here. It should be noted that Antarctic fur seals breed in colonies and are highly philopatric^87^, with distance to the colony often found as a major determinant of female habitat use, even in winter^17,18,20^. In this scenario, the demographic response of the population to improved habitat conditions in distant areas is expected to be delayed, even if males take advantage of the new resources available. This probably explains the extended delay in the recovery of the breeding colonies at the South Georgia and the South Shetland Islands after the cessation of sealing^26,88^.

In conclusion, juvenile, sub-adult and adult male Antarctic fur seals occur year-round in maritime Antarctica and should be incorporated into any ecological model aiming to manage the Antarctic krill population and to manage the fishery in a sustainable way.

## Supplementary Information

Supplementary Information.

## Data Availability

The authors declare that the tracking data from this study will be made publicly available at OBIS-SEAMAP. Correspondence and requests for materials should be addressed to L.C.
