# Supplementary material for: Winter distribution of juvenile and sub-adult male Antarctic fur seals (Arctocephalus gazella) along the western Antarctic Peninsula
Source: Sci Rep. 2021 Nov 15;11:22234. doi: 10.1038/s41598-021-01700-w (PMC8593074; doi:10.1038/s41598-021-01700-w)
Supplement: Supplementary file 1 — Supplementary Information. [file 41598_2021_1700_MOESM1_ESM.pdf]

Supplementary Information

Winter distribution of juvenile and subadult male Antarctic fur seals (*Arctocephalus gazella*) along the western Antarctic Peninsula

David March*, Massimiliano Drago, Manel Gazo, Mariluz Parga, Diego Rita, Luis Cardona

*Corresponding author: David March, email: dmarch@ub.edu. Present address: IRBio and Department of Evolutionary Biology, Ecology and Environmental Science, Faculty of Biology, University of Barcelona, Avinguda Diagonal 643, 08028 Barcelona, Spain.

Contents

Supplementary Tables (Supplementary Table 1)
Supplementary Figures (Supplementary Figures 1-4)

Supplementary Table

Supplementary Table S1. Fitted combinations of the boosted regression tree (BRT) parameters used for model optimization. Results are ordered by cross-validated AUC (CV AUC). Selected parameters for analysis are marked in bold.

Learning rate	Tree complexity	Bag fraction	Num. of trees	AUC	CV AUC	Deviance explained (%)	CV Deviance explained (%)
**0.010**	**5**	**0.5**	**1,050**	**0.95**	**0.88**	**57.5**	**38.0**
0.005	5	0.6	2,050	0.95	0.88	57.1	38.0
0.005	5	0.7	2,300	0.96	0.88	58.3	37.9
0.050	5	0.6	200	0.95	0.88	56.8	37.9
0.005	5	0.5	1,750	0.95	0.88	55.4	38.1
0.001	5	0.5	7,250	0.94	0.88	53.3	37.8
0.010	5	0.6	900	0.95	0.88	55.7	37.9
0.010	5	0.7	850	0.95	0.88	54.9	37.9
0.005	3	0.6	3,200	0.95	0.88	55.0	37.7
0.001	5	0.7	7,950	0.95	0.88	54.1	37.8
0.010	3	0.6	1,600	0.95	0.88	55.0	37.7
0.050	5	0.7	200	0.95	0.88	56.7	38.0
0.010	3	0.7	1,650	0.95	0.88	55.4	37.7
0.001	5	0.6	7,400	0.94	0.88	53.4	37.7
0.050	3	0.6	350	0.95	0.88	56.2	37.6
0.005	3	0.5	2,950	0.94	0.88	54.3	37.7
0.005	3	0.7	3,100	0.94	0.88	54.7	37.8
0.050	3	0.7	300	0.94	0.88	54.3	37.5
0.010	3	0.5	1,350	0.94	0.88	53.4	37.6
0.001	3	0.5	10,000	0.93	0.88	50.3	37.3
0.001	3	0.6	10,000	0.93	0.88	50.1	37.2
0.001	3	0.7	10,000	0.93	0.88	50.0	37.2
0.050	3	0.5	300	0.94	0.88	54.4	36.8
0.050	1	0.6	700	0.90	0.86	43.1	33.0
0.050	1	0.7	800	0.90	0.86	43.6	33.1
0.010	1	0.5	3,300	0.90	0.86	42.9	33.0
0.010	1	0.7	3,750	0.90	0.86	43.3	33.1
0.005	1	0.5	6,550	0.90	0.86	42.9	33.0
0.010	1	0.6	3,800	0.90	0.86	43.6	33.0
0.005	1	0.6	6,550	0.90	0.86	42.7	32.9
0.005	1	0.7	7,050	0.90	0.86	43.0	32.9
0.050	1	0.5	800	0.91	0.86	44.0	32.9
0.001	1	0.5	10,000	0.88	0.85	35.3	29.0
0.001	1	0.6	10,000	0.88	0.85	35.1	28.8
0.001	1	0.7	10,000	0.88	0.85	35.0	28.7
0.050	5	0.5	n.a.	n.a.	n.a.	n.a.	n.a.

Supplementary Figures

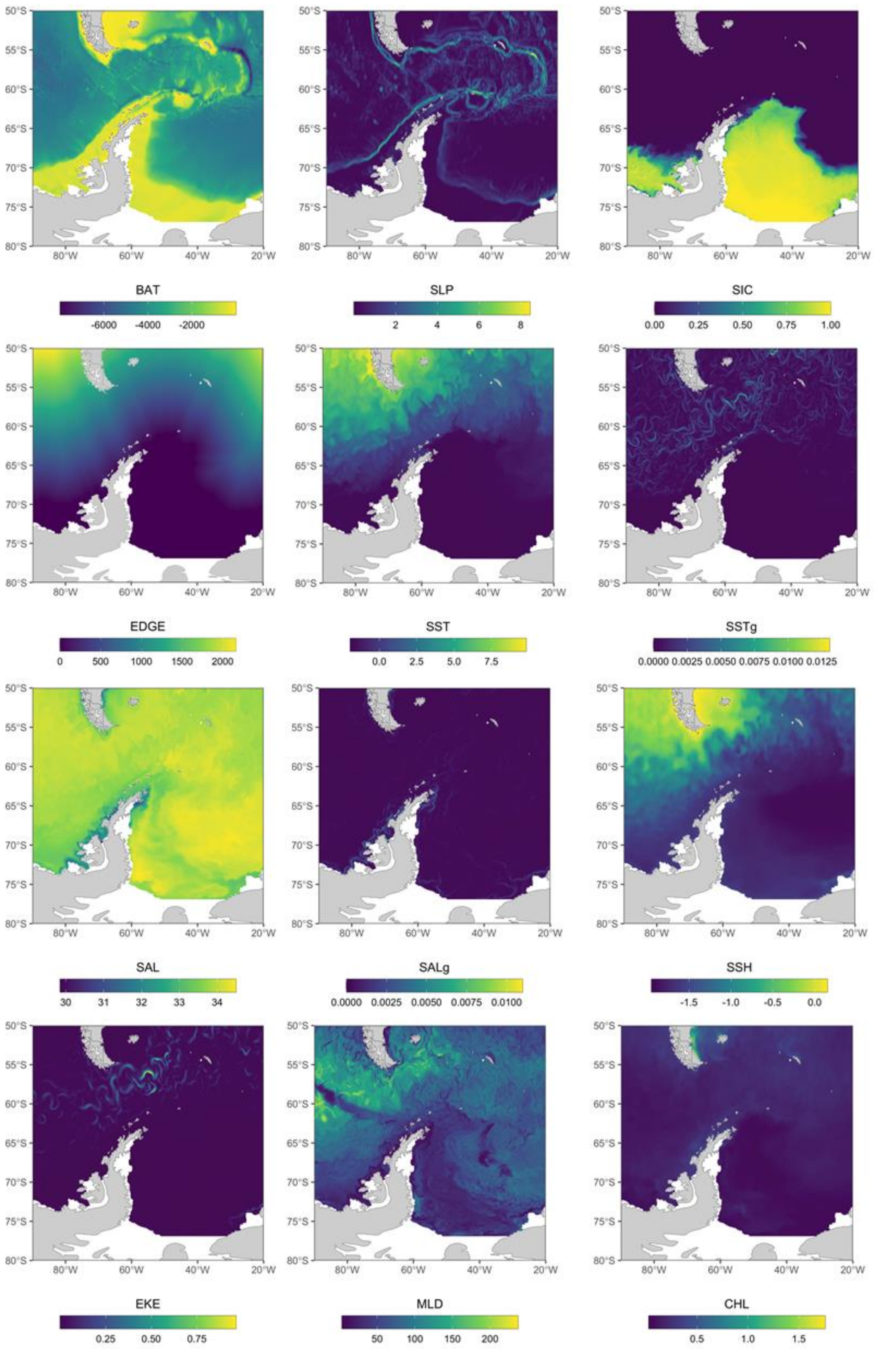

Supplementary Figure S1. Explanatory variables used in the habitat suitability model. Dynamic variables are illustrated using a single day (15th May). Variable acronyms and units are described in Table 2. Maps were generated using R version 4.0.2 (https://www.r-project.org/).

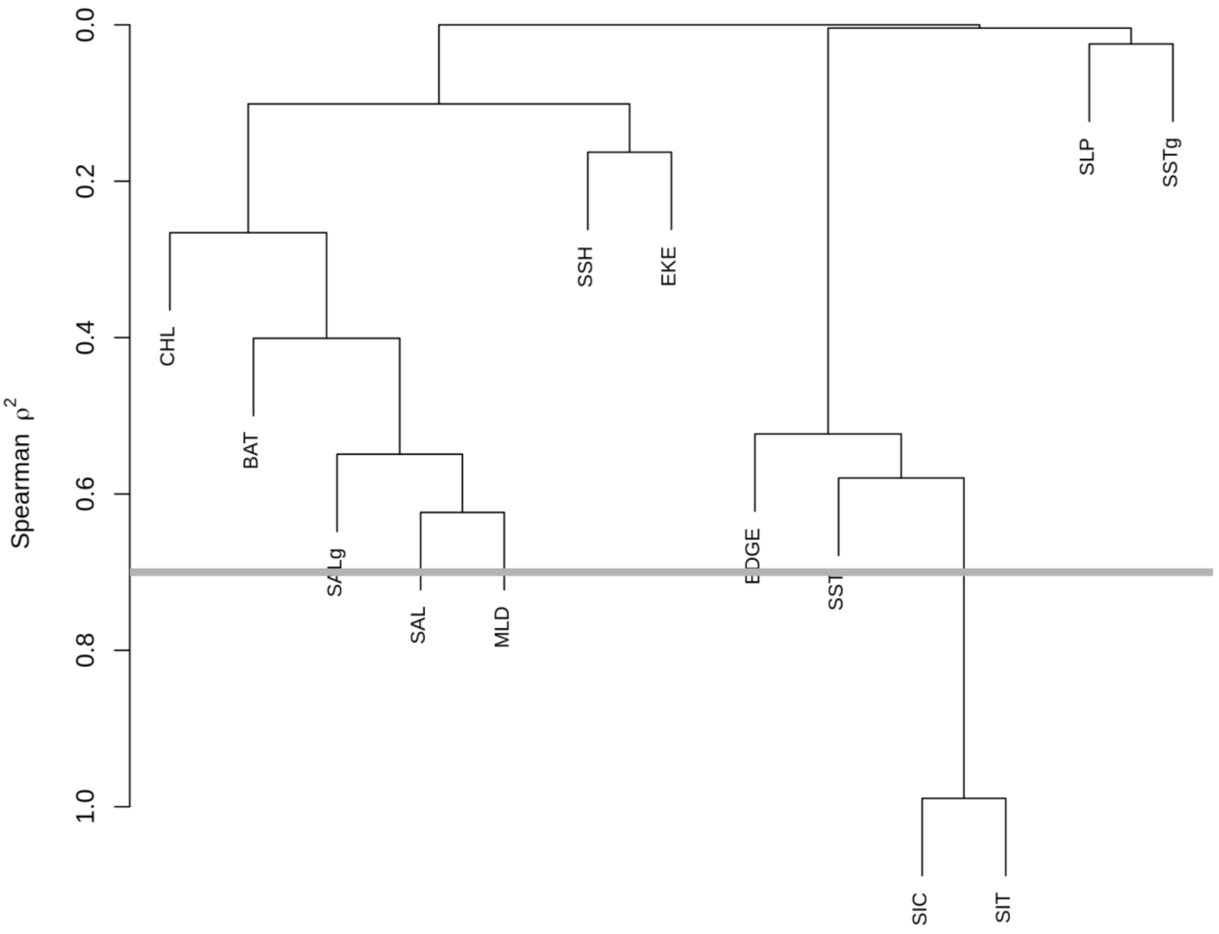

Supplementary Figure S2. Hierarchical cluster on exploratory variables using squared Spearman correlation. The grey line represents the 0.7 threshold used for assessing collinearity. Variable acronyms and units are described in Table 2.

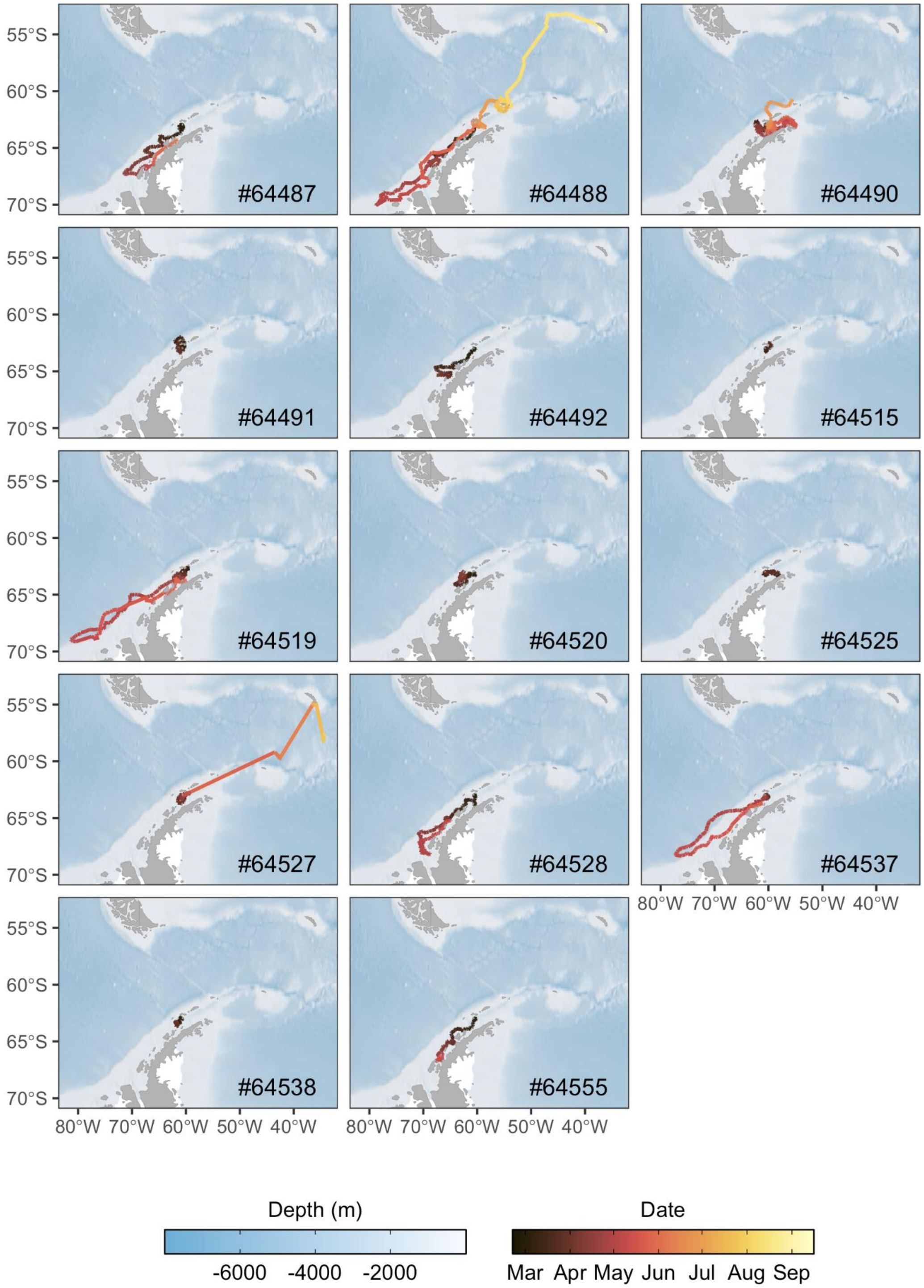

Supplementary Figure S3. Individual tracks of the 14 instrumented juvenile and sub-adult male Antarctic fur seals that transmitted for more than 48 h. Maps were generated using R version 4.0.2 (https://www.r-project.org/).

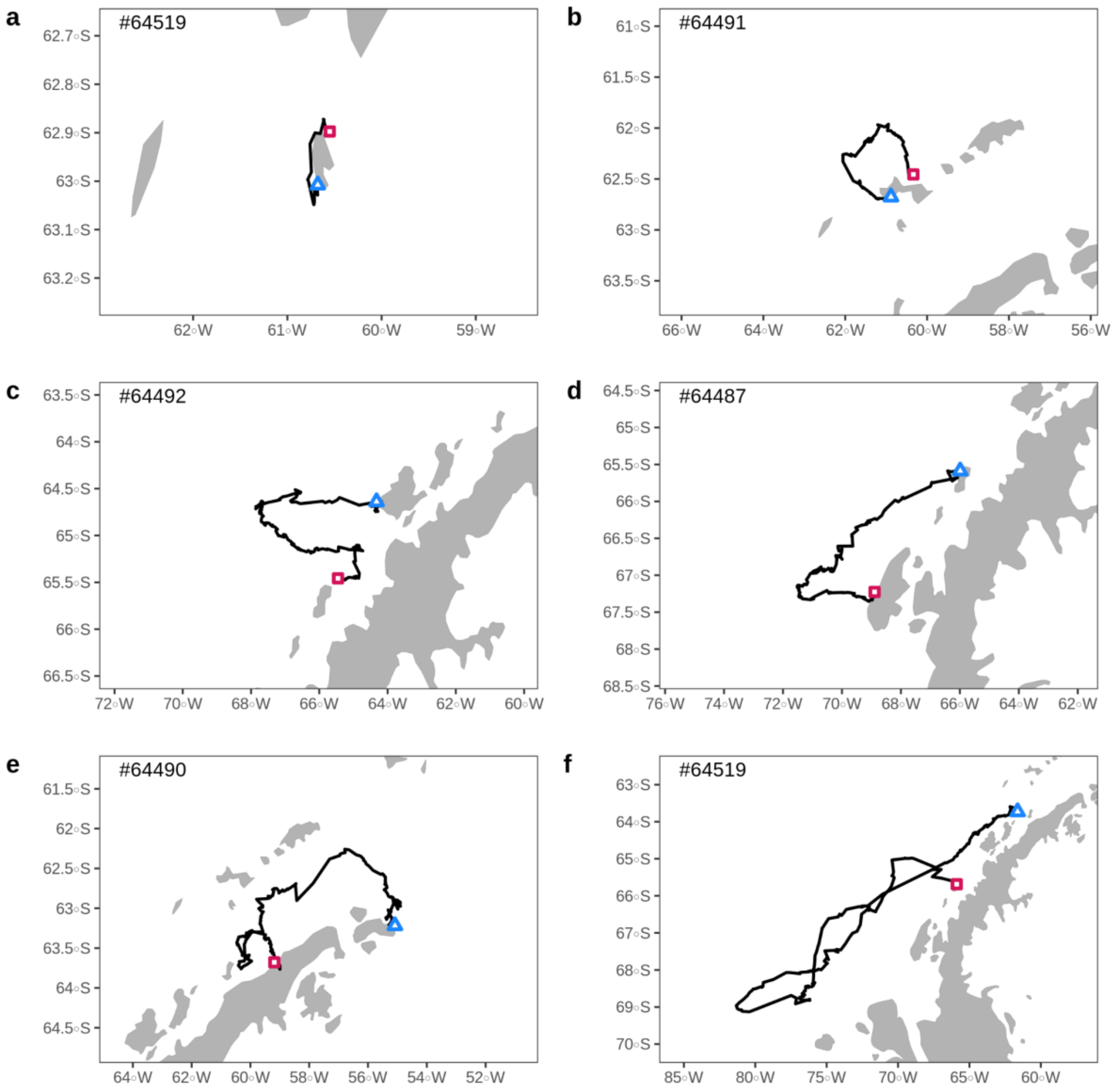

Supplementary Figure S4. Selected foraging trips of juvenile and sub-adult male Antarctic fur seals in early and mid-winter 2019. The blue triangle denotes the departure haul-out and the red square the arrival haul-out of each trip. Round trips, with departure and arrival at the same haul-out or a nearby one in the same island, were uncommon, were observed mainly in February and March and typically lasted one night (panel a: seal #64519, February 28th-March 1st) or a few days (panel b: seal #64491, March 9th-March 13th). As winter advanced, foraging trips became longer and the distance between departure and arrival haul-outs also increased, from tens of kilometers (panel c: seal #64492, March 13th-March 27th) to hundreds of kilometers, both for trips off the South Shetland Islands (panel d: seal #64487, April 4th-April 22nd) and within the Bransfield Strait (panel e: seal #64490, May 15th-May 29th). However, the distance between the departure and arrival haul-outs did not increase necessarily after very long trips (panel f: seal #64519, April 21st-May 21st). Maps were generated using R version 4.0.2 (https://www.r-project.org/).
